# Decision-making towards the end of life – in which way does clinical and ethical reasoning enter QB 13 in palliative medicine?

**DOI:** 10.3205/zma001290

**Published:** 2019-11-15

**Authors:** Bernd Alt-Epping, Alexandra Scherg

**Affiliations:** 1 Universitätsmedizin Göttingen, Klinik für Palliativmedizin, Göttingen, Germany; 2 Deutsche Gesellschaft für Palliativmedizin, AG Bildung, Berlin, Germany

## Background

In the context of an incurable primary disease and in view of finite lifetimes, medical decisions are more explicitly than in other areas of medicine careful considerations and evaluations of different perspectives and in the end are an ethical-normative process. For a highly compromised patient with a far-advanced metastasised tumour who develops severe pneumonia, for example, the question is not only which antibiotic might be suitable for treatment, but also whether an antibiotic might even lead to the desired result and if an antibiotic is (still) appropriate. The clinical and normative criteria that form the basis of these considerations are the particular focus and task of the *Querschnittsbereich Palliativmedizin* (QB13; Cross-Sectional Area of Palliative Medicine). This contribution briefly presents initiatives and projects that (also) aim at teaching and examining students about the processes of clinical and ethical reasoning in decision-making processes in palliative medicine.

## Examples of projects and initiatives

### Chapter “Defining Therapeutic Goals and Limits of Therapy” of the new S3 guideline in palliative medicine

To teach current standards in palliative medicine, the S3 guideline *Palliative Care for Patients with Incurable Cancer* [https://www.leitlinienprogramm-onkologie.de/leitlinien/palliativmedizin/] is a collection of current evidence also for QB13. The subjects of the first part have been complemented by a second part (publishing in 2019) that contains a specific chapter on “Defining Therapeutic Goals and Limits of Therapy” (authors are B. Alt-Epping/DGP; A. Simon/AEM). The focus among other aspects is on the role of patients and their relatives as participants in the decision-making process. The principles of information and patient choice, defining the therapeutic goals, indication of treatment as a process, advance care planning (ACP) and the role of ethical consultation service are presented with as many examples as possible. This chapter is expected to provide many and various options for future student teaching at the faculty by presenting structured and criteria-based information on clinical and ethical reasoning. At Göttingen and Düsseldorf, for example, interprofessional classes are planned in the *problem oriented learning* (POL) format that specifically focus on clinical reasoning. Students of different health and medical professions will be taught together on the basis of this chapter and will together develop the principle of participatory decision-making. They will rehearse this with the help of simulated patients.

#### Decision-making at the end of life as part of the DGIM campaign “Choosing Wisely in Teaching” 

The DGIM-/AWMF campaign “Choosing Wisely” is an initiative to achieve long-lasting improvement in care quality. In this context, diagnostic and therapeutic interventions were identified that are frequently not conducted professionally or were superfluous, or were omitted without reason [1]. These contents were reviewed academically (e.g. diagnostic procedures for a suspected pulmonary artery embolism) for student teaching as a clinical decision-making competency, and examined by using a *key feature* format [2], [3]. QB13 in Göttingen conducted a case vignette on the subject of limits of therapy. This vignette portrays a multi-morbid 82-year-old patient with cardiac insufficiency NYHA IV and recurrent decompensations. After kidney failure, he was to return to a nursing home following a joint decision against renal replacement therapy. The exam questions not only cover pharmacological knowledge, but also further aspects of care and decision-making. This includes questions of how the symptoms can be controlled in the nursing home on the one hand, and normative aspects on the other hand that cover the ethical, legal and medical questions that arise when the shock function of the implanted cardioverter-defibrillator (ICD) is deactivated. This requires distinguishing between killing on request, change in goal of therapy under lacking indication, and an end of therapy based on an expressed patient will. The exam also requires the student to decide for or against deactivating the ICD. This final decision is not part of the evaluation in the exam.

#### OSCEs: Clinical decision-making as criteria-based consideration 

**Figure 1:**
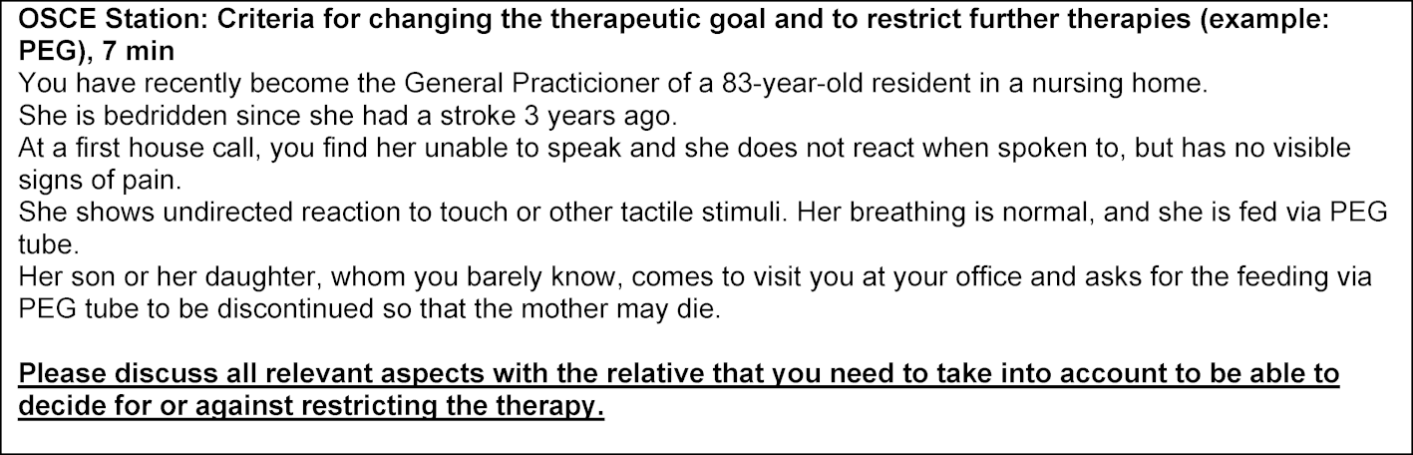
One of the OSCE stations from the Göttingen QB 13 curriculum

Throughout Germany, QB 13 currently focuses on trying out and evaluating formats of Objective Structured Clinical Examination (OSCE). These also include clinical and ethical and ethical aspects of therapeutic decision making, in addition to situations that examine symptom control and communication (see figure 1 (Fig. 1)).

The goal is for students to learn to apply the principles of clinical decision-making in their communications with the patients while becoming aware of the areas of tension that exist between patient´s will and medical indication.

#### “The Virtual Palliative Care Patient”: clinical decision-making in a digital exam 

The coordination office “Evaluative Research” of the Education working Group in the Deutsche Gesellschaft für Palliativmedizin (DGP; German Society for Palliative Medicine) designed a virtual case vignette of a patient with an initial diagnosis of advanced tumour disease. It was piloted at four universities. This showed a very high acceptance of this format, in particular of the video simulation that described the decision-making process, which was considered very helpful.

*“It’s really cool because I felt that I made the decision myself and was then immediately able to see the consequences of my decision in the video sequence! The exam is almost fun in this way because it creates the impression that one is able to master a situation based on one’s very own competence.”* (Reaction of one participant)

## Conclusion

Teaching palliative care in Q13 has always included conveying knowledge about clinical (and ethical) considerations and decision-making about therapy (*clinical reasoning, ethical reasoning*) in the context of incurability and end of life, and also setting this as part of the exams. These aspects complement factual knowledge and skills and are important because they are fundamental in this context.

We chose the 4 example projects to illustrate the direction of our current endeavours at the level of the professional association, for instance, to do justice to the QB13 curricula. These curricula have been implemented formally at almost all German faculties and define the goals of teaching and exams. Students learn through guidelines in teaching and exams to apply these guidelines to clinical problems. By focusing practical and digital exam formats on clinical decision-making, we clarify the relevance these topics have in palliative medicine. These efforts also meet the students’ need to increase their confidence in their interactions with patients at the end of their lives. It also provides the students with the opportunity profoundly analyse death and dying in the context of palliative teaching [4]. 

## Competing interests

The authors declare that they have no competing interests.
